# How does social media use influence the mental health of pancreatic cancer patients: a chain mediating effect of online social support and psychological resilience

**DOI:** 10.3389/fpubh.2023.1166776

**Published:** 2023-06-27

**Authors:** Yi Wang, Sheng Bao, Yubing Chen

**Affiliations:** School of Journalism and Communication, Huaqiao University, Xiamen, China

**Keywords:** pancreatic cancer patients, mental health, social media, mediating effect, online social support, psychological resilience

## Abstract

**Background:**

Pancreatic cancer is an extremely malignant disease that poses a serious threat to the mental health of patients. Many cancer patients now use social media for online social support. However, the impact of social media on mental health is currently inconsistent in the academic community. Therefore, this study aimed to examine the mediating effects of online social support and psychological resilience in the relationship between social media use and mental health of pancreatic cancer patients.

**Methods:**

Four hundred and twenty-five valid questionnaires were collected through convenience sampling. All data were processed using SPSS 26.0 and AMOS 26.0. We examine the influence relationships among latent variables by constructing a structural equation model. Then SPSS Process Macro was used to test the chain mediating effect of the model.

**Results:**

The results showed that (1) anxiety situations occurred in 22.2% of participants (*N* = 94), while the incidence of depression was 20.2% (*N* = 86). (2) Social media use positively influenced online social support (*β* = 0.990, *p* < 0.001), psychological resilience (*β* = 0.504, *p* < 0.001), and mental health (*β* = 0.330, *p* < 0.001); online social support positively influenced psychological resilience (*β* = 0.535, *p* < 0.001) and mental health (*β* = 0.354, *p* < 0.001); psychological resilience significantly and positively influenced mental health (*β* = 0.243, *p* < 0.001). (3) The chain mediating effect of online social support and psychological resilience was significant at 0.253 with a confidence interval of [0.178, 0.340].

**Conclusion:**

Pancreatic cancer patients in China are exposed to a high burden of anxiety and depression, which requires urgent attention. Meanwhile, online social support and psychological resilience played a chain mediating role between social media use and mental health (anxiety and depression), and our results provide new insights and ways to support the mental health improvement of pancreatic cancer patients.

## 1. Introduction

Pancreatic cancer is highly aggressive, metastatic and has a poor prognosis with a five-year survival rate of only 10% (1, 2). Globally, pancreatic cancer is the seventh leading cause of cancer-related death and is expected to overtake colorectal and breast cancer as the second leading cause of cancer-related death by 2030, after lung cancer (3, 4). Public health expenditures related to pancreatic cancer have placed a heavy burden on social, financial and health care systems worldwide in recent years due to the increasing incidence (5). China is one of the countries most severely affected by pancreatic cancer, and according to statistics, 124,000 new cases and 121,000 deaths occurred in China during 2020, posing a serious threat to the lives and health of the citizens (6).

As a major traumatic and stressful event, cancer has been confirmed by numerous studies that the incidence of anxiety and depression in cancer patients is 2–3 times higher than that of the general population, and pancreatic cancer, as a highly malignant gastrointestinal tumor, has a clinical incidence of anxiety and depression of about 16–70%, which is much higher than any other cancer (7–9). Whether in curative or palliative cancer treatment, patients’ anxiety and depression are often overlooked, yet anxiety and depression can have a negative impact on their mental health and quality of life (10, 11). In addition, higher levels of anxiety and depression often indicate longer hospital stays, poorer adherence to treatment, and lower cancer survival rates, and are even strongly associated with an increased risk of suicide in cancer patients (12–14), making this a tough challenge for both public health management and patient disease control. The occurrence of anxiety and depression in cancer patients involves a complex interplay of biological, psychological and social factors, as well as adverse neuropsychiatric effects caused by the use of certain medications during treatment (15). Therefore, the prevention and treatment of anxiety and depression in cancer patients requires multidisciplinary involvement and efforts.

Given the long-term and costly nature of cancer treatment, cancer patients need an effective social support system during the diagnosis and treatment period, as well as the recovery period after discharge, and it is closely related to their mental health and quality of life (16–18). Social support is defined as social resources derived from formal or informal social relationships. The subject of social support in the traditional sense is limited to significant others with considerable intimacy and a certain level of trust, such as family members, peer friends, colleagues, relatives, etc. (19). Meanwhile, the rise of the Internet has attracted an increasing number of cancer patients to join and participate in various social platforms such as communities, hashtags, and health forums based on Internet communication technology, with online communication and interaction as the main functions (20). They use social media to share health information, participate in patient support forums, and seek online social support (21, 22). Online social support breaks the boundaries of offline social support, which is centered on geographic and kinship ties, and allows cancer patients to overcome the limitations of time and space and reach out to a wider cyberspace for social support through social media. Existing studies have confirmed that online social support can also significantly improve the mental health of cancer patients compared with offline social support (23, 24).

Overall, social media use seems to increase patients’ social support, and online social support has a significant effect on cancer patients’ mental health, so does this logic indicate that online social support mediates the relationship between social media use and mental health? Liu et al. (25) followed this line of reasoning and designed a research model to examine the relationship between Facebook use, online social support, and mental health, but found that the mediating effect of online social support could not be established. In contrast, another study of 1,157 social media users in New Zealand found that social media helped users receive online support, which in turn led to mental health benefits for the participants (26).

Additionally, the academic discussion of the potential impact and relevance between social media use and mental health has become a more divisive topic. Numerous studies have highlighted the negative impact of social media use on mental health (27, 28), but there is also literature suggesting that social media use, as a new communication channel, can significantly improve mental health by helping to build connections with relevant others, facilitate social interactions, and maintain relationships (29–31). The established literature has provided conflicting evidence on the association between social media use and mental health, which may be related to factors such as different countries, different sample groups, different intensity of use, and quality of use (32, 33). Therefore, this study responded to the call of Ostic et al. (34) to expand the sample data from different countries and different groups regarding the influencing mechanism of social media use on mental health, hoping to provide new empirical evidence to echo the existing studies. Moreover, it also provided an interdisciplinary view and perspective on how to improve the mental health of pancreatic cancer patients.

Also, this study introduced psychological resilience as a mediating variable. Psychological resilience, as an endogenous resource of individuals, refers to the ability to adapt effectively in the face of adversity, which, for example, helps individuals to overcome difficulties and grow during stressful, potentially traumatic events (35). Studies previously conducted in patients with head and neck cancer (36), endometrial cancer (37), and blood cancer (38) have supported the protective effect of psychological resilience on individual mental health, and high psychological resilience is associated with low depression and anxiety, but few studies in this regard have been conducted involving patients with pancreatic cancer. According to the resilience framework, an individual’s endogenous resilience is activated by external factors, enabling the individual to recover quickly and cope successfully when faced with a traumatic event or great danger, and ultimately resulting in positive mental health outcomes emerge (39). Based on previous work, offline social support may potentially play a role in enhancing psychological resilience (40, 41), but few studies have examined the relationship between online social support and psychological resilience.

**Figure 1:**
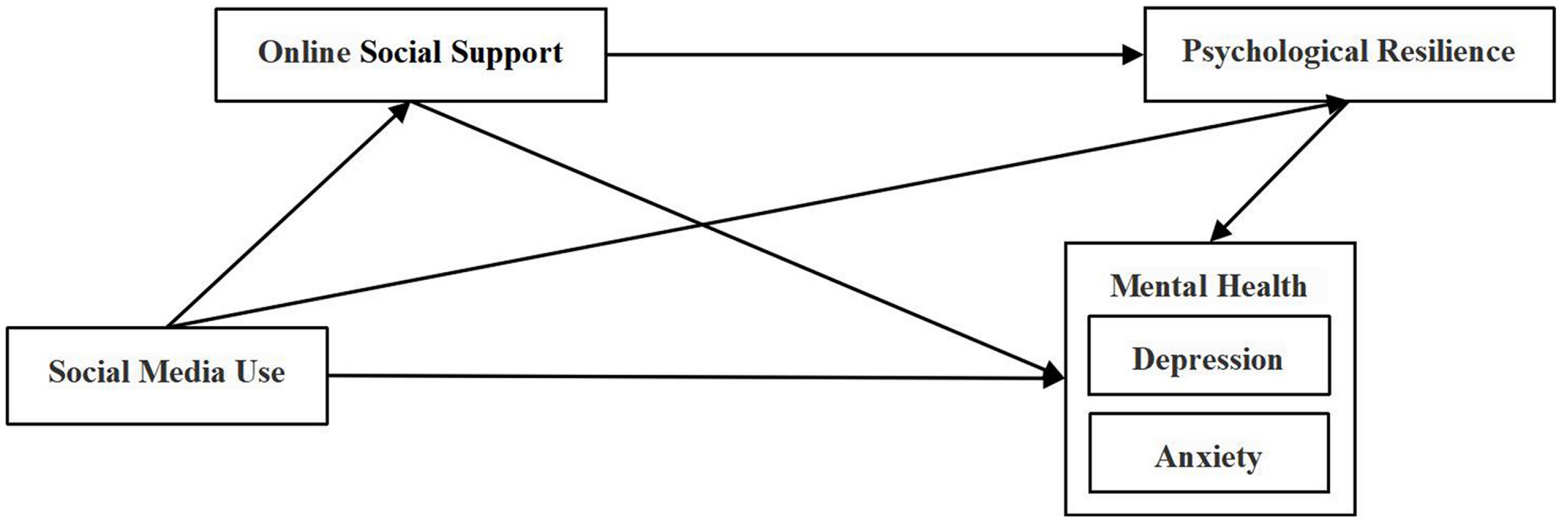
A hypothesized model of the study.

Therefore, this study considered social media use and online social support as external factors, psychological resilience as an internal factor, and depression and anxiety as the final mental health outcomes, to explore whether online social support and psychological resilience play a chain mediating role in the influencing mechanism of social media use on mental health among pancreatic cancer patients. The hypothesized model of this study was shown in Figure 1.

## 2. Materials and methods

### 2.1. Data source and sampling

In this study, participants were recruited through a convenience sampling method. Recruitment information was posted on the mainstream social media platforms (Weibo, Douban, Zhihu, etc.) in China to collect online data during October 1, 2022 to December 1, 2022. Online surveys have been recognized as a quick and convenient but effective method of data collection (42). The study was approved by the Ethics Committee of the School of Journalism and Communication, Huaqiao University. Before completing the closed-ended questionnaire, each respondent was informed of the purpose of the study and assured that their participation would be voluntary, strictly confidential, and anonymous. Informed consent was also obtained from each participant. Then they were invited to complete the online questionnaire using the “WJX”^1^ platform, which is currently the largest online questionnaire platform in China.

### 2.2. Data collection

**Figure 2:**
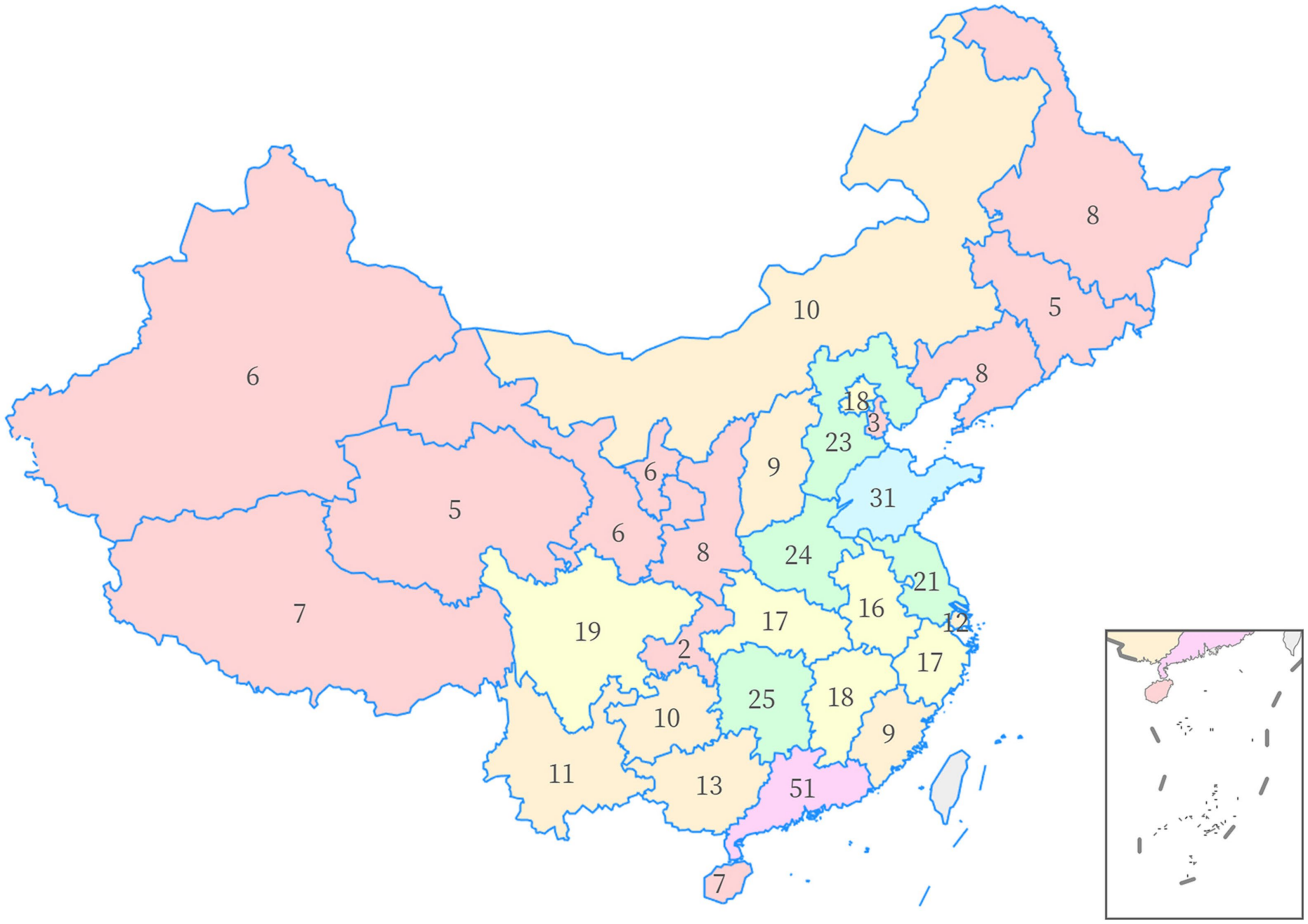
Geographical distribution of samples.

The sample size was determined using a 1:10 measure for each observed variable (43), and the questionnaire delivered in this study had 39 observed variables, indicating that a minimum sample size of 390 needed to be collected. A total of 463 online questionnaires were finally collected, and after excluding 38 invalid samples (insufficient response time, repeated completion by the same IP address, etc.), 425 valid samples were finally included, with an effective rate of 91.8%, and the sample size could meet the research needs. The data of pancreatic cancer patients were from 31 provinces (including autonomous regions and municipalities) in China, and the sample distribution of each province was shown in Figure 2, which basically corresponded to the population distribution characteristics of dense population in southeastern China and sparse population in northwestern China (44).

### 2.3. Measures

The questionnaire consisted of five sections: demographic information, social media use, online social support, psychological resilience, and mental health. To ensure the reliability of the questionnaire, a strict translation-back translation procedure was used in this study, by translating the English measure items into Chinese and then back-translating them to ensure that the statements were accurate and unambiguous. The main variables are measured as follows:

#### 2.3.1. Social media use

This section was adapted from Karikari et al. (45) to measure patients’ social media use with five items, including “Social media is an essential part of my daily life” “I browse health information and health knowledge on social media every day” etc. These items were measured on a five-point Likert scale ranging from 0 (strongly disagree) to 4 (strongly agree) (Cronbach’s *α* = 0.947, AVE = 0.782, CR = 0.947).

#### 2.3.2. Online social support

The measure of online social support was adapted from Nick et al. (46) and consisted of 10 items and four dimensions: emotional support, peer support, informational support, and instrumental support, using a five-point Likert scale ranging from 0 (never) to 4 (a lot). These items include “Social media users show concern and compassion for me” “I have access to useful treatment, medical and health information on social media when I need it” etc. (Cronbach’s *α* = 0.952, AVE = 0.802, CR = 0.953).

#### 2.3.3. Psychological resilience

Psychological resilience was assessed using a measure developed by Campbell et al. (47). The measure consisted of 10 items, such as “I do not get discouraged easily by failure” and “I do not give up easily when things do not look so hopeful.” These items were measured on a five-point Likert scale ranging from 0 (strongly disagree) to 4 (strongly agree) (Cronbach’s *α* = 0.952, AVE = 0.799, CR = 0.952).

#### 2.3.4. Mental health

We used the Chinese version of the Hospital Anxiety and Depression Scale, originally developed by Zigmond and Snaith (48), and the Chinese version of the HAD scale has been validated to have good reliability and validity (49). The scale includes an anxiety (HADS-A) and an depression (HADS-D) sub-scale, each consisting of seven items rated on a four-point Likert scale (0–3), so that each sub-scale can be scored up to a maximum of 21 points. The scale was designed to focus only on participants’ psychiatric symptoms by excluding organic disease factors (e.g., dizziness or headaches). A reverse scoring method was used, meaning that the higher the score, the higher the level of mental health and the lower the level of anxiety and depression (Cronbach’s *α* = 0.975, AVE = 0.799, CR = 0.976).

When the Cronbach’s α is >0.9, the intrinsic reliability of the questionnaire measurement items can be considered excellent. Also, the convergent validity and discriminant validity of the measurement items were evaluated using the composite reliability (CR) and the average variance extracted (AVE). It is generally required that the CR of the latent variables should be >0.7, as well as the AVE should be >0.5 (50). All four latent variables included in this study (SMU, OSS, PR, and MH) met the recommended requirements, indicating that the measures had good reliability and validity.

### 2.4. Statistical analysis

All data were processed by SPSS 26.0 (SPSS, Inc., Chicago, IL, United States) and AMOS 26.0 (Amos Development Corporation). First, to test the differences in mental health across demographic characteristics, independent sample *t*-tests, one-way ANOVA, and Pearson correlation analyses were conducted. Second, Pearson correlations analyses were also performed to evaluate the associations between the main variables. Then, a structural equation model was constructed to test the goodness of fit of the model and the effect relationships among the latent variables. Finally, the SPSS PROCESS Macro, developed by Preacher and Haye (51), was used to test the chain mediating effect of the model.

## 3. Results

### 3.1. Demographic characteristics of participants

The demographic characteristics of the participants were shown in Table 1, which in this study mainly included their gender, age, educational background, marital status, monthly income, and tumor staging. The average age of the 425 valid participants was 42.79. The majority of participants had a junior college degree or above, with 17.4% having a junior college degree, 26.4% having a bachelor’s degree, and 14.6% having a postgraduate degree and above. Regarding marital status, 51.1% of the patients were married. Monthly income showed that those with a monthly income of CNY 4001–6,000 accounted for a relatively large proportion, 30.4%. In terms of tumor staging, most of the samples had stage III cancer, with 58.8%. All participants denied previous diagnoses of anxiety and depression. overall, the sample met the required characteristics of the target population in our study

**Table 1:** Demographic characteristics of the samples.

Characteristic	Demographic information	Frequency	%
Gender	Male	183	43.1
	Female	242	56.9
	Others	0	0
Age	Mean: 42.79, SD: 9.499		
Education	Primary school and below	15	3.5
	Junior High School	65	15.3
	Senior High School	97	22.8
	Junior College	74	17.4
	Bachelor degree	112	26.4
	Postgraduate degree and above	62	14.6
Marital status	Unmarried	59	13.9
	Married	217	51.1
	Divorced	120	28.2
	Widowed	29	6.8
	Others	0	0
Monthly income (CNY)	≤2,000	28	6.6
	2,001–4,000	77	18.1
	4,001–6,000	129	30.4
	6,001–8,000	119	28
	>8,000	72	16.9
Tumor staging	Stage 0	0	0
	Stage I	0	0
	Stage II	96	22.6
	Stage III	250	58.8
	Stage IV	79	18.6

“CNY” refers to the China Yuan.

### 3.2. Differential testing of demographic characteristics

Table 2 showed the differences in mental health under different demographic characteristics.

**Table 2:** Differential testing of demographic characteristics in mental health (*N* = 425).

Characteristic	Demographic information	Mean	SD	*t*, *F*, or *r*	*p*-value
Gender*	Male	2.2	0.66	−2.34	0.001
	Female	2.44	0.55		
Age*		42.79	9.49	−0.27	0.001
Education*	Primary school and below (a)	1.89	0.8	5.69	0.016
	Junior High School (b)	2.17	0.62	a < b < c	
	Senior High School	2.24	0.61		
	Junior College	2.48	0.5		
	Bachelor degree	2.36	0.63		
	Postgraduate degree and above (c)	2.55	0.5		
Marital status*	Unmarried (a)	2.55	0.47	5.17	0.03
	Married	2.37	0.59	c < b < a	
	Divorced (b)	2.23	0.64		
	Widowed (c)	2.1	0.73		
Monthly income (CNY)*	≤2,000	2.46	0.53	6.87	0.001
	2,001–4,000 (a)	2.17	0.61	a < b < c < d	
	4,001–6,000 (b)	2.24	0.61		
	6,001–8,000 (c)	2.34	0.67		
	>8,000 (d)	2.63	0.41		
Tumor staging*	Stage II (a)	2.61	0.44	15.32	0.001
	Stage III (b)	2.34	0.62	c < b < a	
	Stage IV (c)	2.11	0.62		

Variables with “*” indicate the heterogeneity of variance, using the Welch method.

SD is the standard deviation.

The female group in the sample had a higher level of mental health compared to males with a significant difference (*t* = −2.34, *p* < 0.001). And age and mental health were negatively correlated (*r* = −0.27, *p* < 0.001). In the case that the one-way ANOVA results were significant, *post-hoc* tests were performed for a two-by-two comparison to check the differences between each group. And the results revealed that participants with postgraduate degree and above had a higher level of mental health than those with a primary school degree and below and those with a junior high school degree. Furthermore, we found significant differences in the relationship between marital status and mental health, specifically higher levels of mental health among unmarried participants than divorced and widowed participants (*F* = 5.17, *p* = 0.03).

We also noticed that the mental health status of pancreatic cancer patients differed by monthly income, and participants with monthly income >8,000 had significantly better mental health than other groups (*F* = 6.87, *p* < 0.001). And cancer staging was also associated with participants’ mental health (*F* = 15.32, *p* < 0.001), participants with stage II had significantly higher levels of mental health than those with stage III and stage IV. In conclusion, unmarried women with postgraduate education or above, and participants with monthly income >8,000 and stage II cancer showed the highest level of mental health.

In addition, we calculated the incidence of depression and anxiety among the participants (see Table 3). According to the criteria of Zigmond and Snaith, cumulative scores of 0–7 were classified as no cases, 8–10 as doubtful cases, and 11–21 as cases (48). For this study, we adopted a reverse scoring method, so that 0–10 were classified as cases, 11–13 as doubtful cases and 14–21 as normal, and finally we found that the incidence of anxiety was 22.2% (*N* = 94) and depression was 20.2% (*N* = 86) among the participants.

**Table 3:** Anxiety and depression of participants.

	Anxiety category			Depression category		
	No cases	Doubtful cases	Cases	No cases	Doubtful cases	Cases
*N*	293	38	94	286	53	86
*n* (%)	68.90%	8.90%	22.20%	67.30%	12.50%	20.20%

### 3.3. Test of common method biases

Common method bias is the error caused by the same measurement environment and the same data source. Since the data in this study were obtained from questionnaires, common method bias may exist. To test for the existence of common method bias, this study used Harman’s single-factor test to analyze all items of the key variables. Since the amount of variation explained by the first factor was 25.3%, which was below the standard threshold of 40%. This indicated that there was no serious common method bias in this study and the data could be analyzed in the next step.

### 3.4. Structural equation model test

Based on the hypothesized model, we constructed a structural equation model using Amos 26 to examine the pathways of influence among social media use, online social support, psychological resilience, and mental health among patients with pancreatic cancer.

First, we assessed the fit of the hypothesized model, and the following criteria suggested by Hooper et al. (52) were adopted for the model fit: (1) The chi-square to degree of freedom ratio (*x*^2^/df), should be between 1 and 5; (2) The root means square error of approximation (RMSEA), should be <0.08; (3) The standardized root means square residual (SRMR), should be <0.08; (4) Tucker-Lewis index (TLI), should be >0.95; (5) The normed fit index (NFI), should be >0.9; (6) Comparative fit index (CFI), should be >0.9. The detailed model fitting indexes were given in Table 4, and all the indexes have met the suggested criteria, indicating that the model fit is good.

**Table 4:** Indexes of model fit.

Index	*x*^2^/df	RMSEA	SRMR	TLI	NFI	CFI
Observed value	3.212	0.072	0.02	0.96	0.948	0.964
Ideal value	<5	<0.08	<0.08	>0.95	>0.9	>0.9

RMSEA, root means square error of approximation; SRMR, standardized root means square residual; TLI, Tucker-Lewis index; NFI, normed fit index; CFI, comparative fit index.

**Figure 3:**
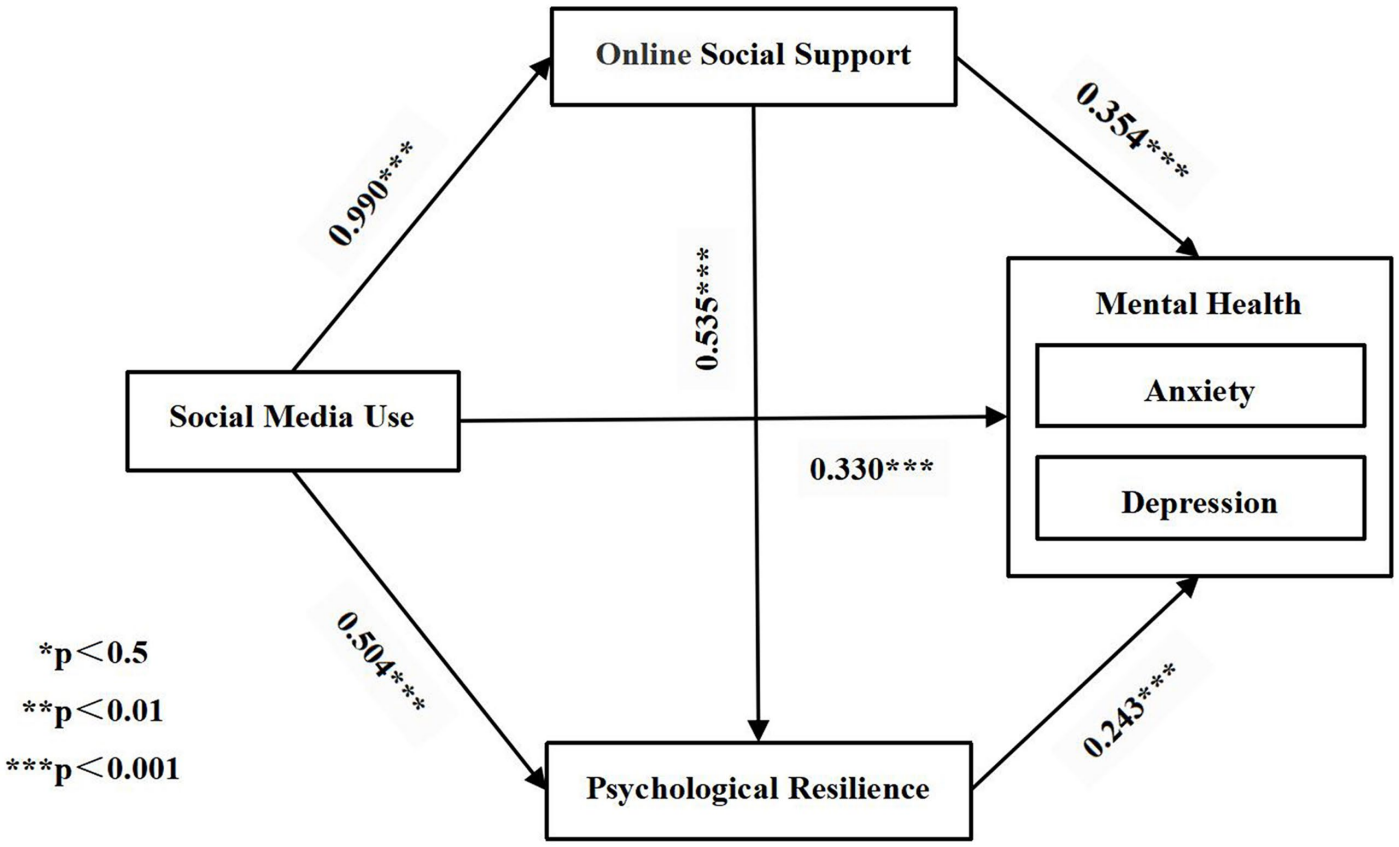
Structural equation model path coefficients.

There were six paths in this study, and the paths of the structural equation model were analyzed in Amos26. The final path testing results of the model were shown in Figure 3 and Table 5. The results showed that all six paths were supported and at the significant level of *p* value < 0.001. Social media use positively influenced online social support (*β* = 0.990, *p* < 0.001), psychological resilience (*β* = 0.504, *p* < 0.001), and mental health (*β* = 0.330, *p* < 0.001). And online social support positively influenced psychological resilience (*β* = 0.535, *p* < 0.001) and mental health (*β* = 0.354, *p* < 0.001). We also found that psychological resilience had a positive effect on mental health (*β* = 0.243, *p* < 0.001).

**Table 5:** Path testing results.

Model paths	Path coefficients	Standardized estimate	*p*-values
	(β)		
Social media use → Online social support	0.99	0.033	<0.001
Social media use → Psychological resilience	0.504	0.042	<0.001
Social media use → Mental health	0.33	0.04	<0.001
Online social support → Psychological resilience	0.535	0.043	<0.001
Online social support → Mental health	0.354	0.039	<0.001
Psychological resilience → Mental health	0.243	0.035	<0.001

### 3.5. Chain mediating effect test

The path test of the structural equation model verified the potential relationships among the variables in this study, and then we tested the chain mediating effects of the model through the SPSS PROCESS Macro. We estimated the 95% confidence intervals of the mediating effect with 5,000 resamples, and when the confidence interval excluded 0, the mediating effect was verified, as shown in Table 6. The mediating effect of “social media use → online social support → mental health” was 0.304, 30.47% of the total effect, with a 95% confidence interval of [0.204, 0.413], indicating a significant mediating effect of online social support. And the mediating effect of “social media use → psychological resilience → mental health” was 0.202, 20.25% of the total effect, with a 95% confidence interval of [0.131, 0.288], indicating a significant mediating effect of psychological resilience. The chain mediating effect of “social media use → online social support → psychological resilience → mental health” was 0.253, accounting for 25.35% of the total effect, with a confidence interval of [0.178, 0.340], suggesting that a significant chain mediating effect of online social support and psychological resilience was verified.

**Table 6:** Effects and 95% confidence intervals for model.

Model paths	Indirect effect value	Boot SE	Boot CI lower	Boot CI upper	Relative mediation effect
Total mediation effect	0.759	0.049	0.662	0.858	76.07%
Social media use → Online social support → Mental health	0.304	0.053	0.204	0.413	30.47%
Social media use → Psychological resilience → Mental health	0.202	0.039	0.131	0.288	20.25%
Social media use → Online social support → Psychological resilience → Mental health	0.253	0.042	0.178	0.34	25.35%

## 4. Discussion

### 4.1. Mental health of pancreatic cancer patients

Our study revealed a worrying fact that among 425 participants, the incidence of anxiety was 22.2% (*N* = 94) and depression was 20.2% (*N* = 86), which were higher than previous surveys on the anxiety and depression status of pancreatic cancer patients in the United States and Australia (53, 54), suggesting that pancreatic cancer patients in China are currently facing a higher burden of anxiety and depression and need urgent attention. Moreover, the results of mental health differences under demographic variables indicated that the anxiety and depression conditions of pancreatic cancer patients were closely related to gender, age, education, income, marital status, and cancer staging, and that men of older age with lower education, lower income, and divorced or widowed marital status may face a more severe burden of anxiety and depression, a finding similar to previous studies (55, 56).

Compared with high-income and high-education groups, low-income and low-education groups face more severe living difficulties and economic pressures (57), as well as lower mental health literacy and medical knowledge (58). The high burden of cancer treatment and the financial loss due to loss of work and salary during the treatment make patients fall into the “poverty trap,” and insufficient awareness of depression and anxiety prevents patients from adopting science-based psychological interventions and neglecting the need to seek professional help. Meanwhile, men who are widowed or divorced at an older age are likely to lack adequate social networks and partner support. However, masculinity as an ingrained cultural psychology, makes some men reluctant to reveal their vulnerability, hide their inner thoughts and emotions (59, 60), and enter into a silent crisis of mental health status.

Accordingly, the mental health of pancreatic cancer patients demands immediate attention, and the mental health risks faced by older men with low income, low education, widowed or divorced are even higher, making them the top priority for health management and psychological screening. Furthermore, the reimbursement rate of medical insurance and medical subsidy policy should be appropriately tilted to cancer patients with low-income, so as to prevent “poverty due to illness” and reduce the financial burden of patients.

Nowadays, the United States, Australia and other developed countries are increasingly paying attention to the mental health problems and psychological intervention management of cancer patients, screening cancer patients for anxiety and depression symptoms in accordance with the guidelines, and providing stepped care to patients according to the severity of their symptoms (61, 62). Unfortunately, China still lacks standardized mental health screening guidelines for cancer patients. In order to ensure standardization and rationality of relevant interventions, there is an urgent need to combine research findings and relevant management guidelines from developed countries to construct management guidelines that fit the current situation of mental health in Chinese cancer patients. For example, mental health should be clinically monitored as a vital sign, and patients should be assessed at key points of change in their condition, such as diagnosis, initiation of clinical treatment, review, disease recurrence and metastasis, transition from curative treatment to palliative care or hospice, and end of life. Second, electronic patient-reported outcomes (ePRO) have become a hot spot for both current applications and research, and are gaining prominence in mental health screening for cancer patients (63). The benefits of ePRO include ease of use with patients, time savings, and ease of data management. Incorporating ePRO screening into a clinical routine helps focus doctor-patient communication on the patient’s current mental health crisis and guides the clinician to make quicker clinical judgments. Finally, we need to establish multidisciplinary to holistic integrative management (MDT to HIM) teams that include oncologists, nurses, psychologists, psychiatrists, social workers, family members, and other patient advocates. This will provide different levels of support for the different mental health problems that patients present with. Classification and orderly triage of patients based on screening results can reduce the number of patients who blindly seek help during their visits and medical treatment.

### 4.2. Positive effects of social media use on mental health

Our study found that social media use not only indirectly affected the mental health of pancreatic cancer patients through the chain mediating effects of online social support and psychological resilience, but that social media use may also have a direct positive effect on mental health. This finding echoed the existing studies, indicating that social media had a positive impact on mental health in pancreatic cancer patients as well. The Centers for Disease Control and Prevention suggests that social media can serve as an essential platform for cancer prevention, early detection, treatment and health improvement of cancer patients (64). Previous relevant studies have also provided empirical evidence for the role of social media in cancer treatment and mental health management. When cancer patients use social media for more health-related activities, they are more motivated to engage in self-care and health management (65). More specifically, they may actively seek information to understand their emotional load and implement effective strategies to cope with the psychological burden and reduce the risk of developing further severe anxiety and depression during cancer care (66, 67).

The World Health Organization reports that low and middle-income countries face a significant “mental health gap” compared to high-income countries, with a large gap between those who need mental health care and the professionals who provide such services (68). According to statistics from China’s National Health Commission, by the end of 2021, the number of psychiatrists in China was only 64,000 (69), with an average of only one professional doctor for every 25,000 people to provide mental health services for a huge population of 1.412 billion. In the face of such a dramatic disparity, it is clear that traditional forms of mental health services (offline face-to-face personalized assessments and medical interventions) are unsustainable for pancreatic cancer patients, but social media offers a new and alternative idea. Online psychological interventions based on social media have been shown to be effective in many countries (70, 71), even under the guidance of a para-professional (72).

Therefore, using social media to support pancreatic cancer patient’s mental health is promising. First of all, the government and the healthcare sector can take the lead and cooperate with medical personnel in the fields of oncology and psychiatry, medical practitioners and psychological counselors. Taking advantage of social media’s convenience, freedom, wide coverage and low cost, we can build a social media platform that meets oncology patients’ needs and provides good support services. This platform may serve as an online venue for mental health education, counseling, and psychotherapy interventions for cancer survivors. Second, paraprofessionals (e.g., lay counselors, community workers, nurses, and other clinical staff) should also be trained to join the ranks of online psycho-education and therapeutic interventions to benefit a wide range of patient populations through social media health science and remote assistance. It has also been clinically proven that Internet-based cognitive behavioral therapy (ICBT) is effective (73, 74). However, ICBT is not yet popular in China, so in the future, we need to develop ICBT based on pancreatic cancer groups and relevant online platforms as part of social media’s functions to facilitate patients’ use. Third, to encourage active patient participation, online support groups and social media discussion forums should be developed. As a powerful two-way communication tool, social media allows patients to take the initiative to seek a wider variety of treatment and medical advice, and to receive peer support by participating in online support groups. Through expressive writing on social media, patients can vent extreme emotions such as fear and anger, allowing them to express their feelings, share information, and be supported online (75, 76). Finally, advanced pancreatic cancer patients face higher mental health risks, but the technical complexity of smartphones is a barrier to their use of social media. Future social media-based mental health interventions should be developed in an older adult-friendly version with a simpler interface and more convenient operation to meet the needs of the older adult. Assistive features such as screen reading should also be made available so that the mental health of advanced patients with pancreatic cancer can also benefit from easier and faster access to social media.

### 4.3. Chain mediating effects of online social support and psychological resilience

The test of mediating effects revealed that online social support and psychological resilience not only played independent mediating roles among the impact of social media use on the mental health, but also there was a chain mediating effect of them. A past survey involving primary healthcare workers during the pandemic demonstrated that offline social support had a significant impact in enhancing participants’ psychological resilience and ultimately improved mental health outcomes (anxiety and depression) through a chain mediating effect (77). Our results were in line with it, and online social support also had a similar effect in our study.

This finding could be explained by the Buffering Hypothesis proposed by Cohen et al. This hypothesis suggests that offline social support acts as a buffer between the effects of stressful events on mental health and protect individuals from the negative effects of psychological distress to some extent (78). Also, previous literature has suggested that psychological resilience as an intrinsic personal resource can also buffer mental health crises caused by cancer (79). When it comes to major diseases such as pancreatic cancer, institutionalized social security (e.g., health insurance) is not sufficient, and traditional social support sources (e.g., families, relatives, and friends) may not be able to empathize with the patient’s situation. Social media has become a new approach for pancreatic cancer patients to seek online social support when offline social support is insufficient or absent. Established work has pointed out that social media can not only provide emotional and informational support for cancer patients, but also complement material support that is insufficient or absent in traditional social support (24, 80). At the level of emotional and informational support, offline social support systems have difficulty understanding all the physical and psychological suffering caused by the survival anxiety and treatment that patients face, as well as providing the healthcare information that they need. In social media platforms such as online cancer communities, patients are able to gain space for emotional catharsis and receive valuable treatment information together with encouraging and inclusive emotional experiences in the messages and comments of their peers, thus promoting their mental health (81). Moreover, with insufficient social security, cancer patients have to face the financial hardship of high treatment costs, and friends, relatives and acquaintances probably are unable to provide adequate material support. Social media gives patients the opportunity to relieve financial stress by providing extra material security through streaming reward, cash transfer, and online crowdfunding (82), which helps those suffering from misfortune to get through difficult times in some ways.

Our findings suggest that online social support, which stimulates endogenous resilience, significantly affects patients’ mental health. Given these findings, we believe that interventions aimed at increasing online social support and social media resilience may be effective in improving the mental health of pancreatic cancer patients. First, healthcare professionals, oncologists, psychiatrists, nurses and social workers should be encouraged to join the social media platform. In this way, a four-level social media intervention matrix with relevant medical departments-hospital-society-patients can be formed to provide participants with favorable online social support, including medical support, information support, emotional support, and companionship support. In addition, patients are encouraged to proactively participate in social media support groups and online group chats, and they are also supported in sharing their cancer journey through video and text in content communities. Previous studies have confirmed that online video creation can help stimulate positive emotions (hope, humor, etc.), release negative emotions (fear, sadness, etc.), and increase peer camaraderie and a sense of community belonging among cancer patients (83). By writing about their experiences, patients are able to regain a sense of control over their lives, and this process also gives them new self-efficacy and psychological resilience (84). Also, cancer patients enrich their social support network through active participation and creation. They turn online social support into inner strength and greater mental resilience, and ultimately, improvement in their mental health.

However, it should be emphasized that online social support and offline social support are not mutually exclusive. Online social support can, to a certain extent, complement the absence of offline social support, but online social support may show transient and unstable traits (85), and it is also difficult to fully replace the offline social support provided by families in real life, such as long-term daily care. Therefore, social policy makers need to build a more comprehensive and balanced social security system to provide patients with a more diversified online and offline social support network in coping with serious diseases such as pancreatic cancer. This support network consists of a combination of social support from the formal system, the public sphere, and the private sphere (including the social security system, families, relatives and friends, medical professional groups, social workers, social media, etc.) to provide patients with more comprehensive protection and support, improve their mental resilience and mental health, and ultimately improve their survival rate and quality of life.

## 5. Conclusion

This study provides the evidence of a chain mediating effect of online social support and psychological resilience between social media use and mental health in pancreatic cancer patients. In our collected sample (*N* = 425), 22.2% of participants (*N* = 94) had anxiety symptoms, and the incidence of depression was 20.2% (*N* = 86), suggesting the high burden of anxiety and depression among pancreatic cancer patients in China and the urgent need to develop a screening system and intervention policy for their mental health in accordance with the Chinese context. Our data analysis also provided new insights and ways to support the improvement of mental health in pancreatic cancer patients, indicating that online social support and psychological resilience play a chain mediating role between social media use and mental health (anxiety and depression).

## 6. Limitations and future work

However, there are also limitations in this study. First, this study used a cross-sectional design and therefore was not sufficient to infer direct causality from the findings. Future longitudinal studies and controlled experimental methods could be adopted to explore the causality between social media use and mental health. Second, we could not distinguish differences in mental health among patients with different treatments (e.g., radical resection, adjuvant chemotherapy, etc.) in terms of control variables. Future studies could further explore the mental health of patients with different treatments and identify differences using the TNM tumor staging. Third, the data were collected through recruitment information posted on Internet platforms, so there might be sampling bias since people with better Internet access were more likely to be recruited. Fourth, social media use is a relatively broad concept. We did not distinguish among different types of social media platforms, quality of social media use, passive and active use, etc. More diverse dimensions for measuring social media use could be considered in the future. Last, mental health is also a complex concept, and our study only included anxiety and depression as assessment indicators. Future studies could also include various indicators such as loneliness, stress, subjective well-being, and life satisfaction to measure patients’ mental health in a comprehensive manner.

## Data availability statement

The original contributions presented in the study are included in the article/supplementary material, further inquiries can be directed to the corresponding author.

## Ethics statement

The studies involving human participants were reviewed and approved by Ethics Committee of the School of Journalism and Communication, Huaqiao University. The patients/participants provided their written informed consent to participate in this study.

## Author contributions

YW and SB contributed to conception and design of the study. SB wrote the first draft of the manuscript, designed the model and questionnaires, and performed the data analysis. YW collected the data. YC revised and edited the manuscript. All authors contributed to the article and approved the submitted version.

## Conflict of interest

The authors declare that the research was conducted in the absence of any commercial or financial relationships that could be construed as a potential conflict of interest.

## Publisher’s note

All claims expressed in this article are solely those of the authors and do not necessarily represent those of their affiliated organizations, or those of the publisher, the editors and the reviewers. Any product that may be evaluated in this article, or claim that may be made by its manufacturer, is not guaranteed or endorsed by the publisher.

## Footnotes

^1^ www.wjx.cn
